# Minimum dietary diversity and associated factors among children under the age of five attending public health facilities in Wolaita Soddo town, Southern Ethiopia, 2021: a cross-sectional study

**DOI:** 10.1186/s12889-022-14861-8

**Published:** 2022-12-16

**Authors:** Fikre Moga Lencha, Zenebe Jebero Zaza, Lankamo Ena Digesa, Tegegn Mulatu Ayana

**Affiliations:** College of Medicine and Health Sciences, Arba Minch University, P.O. Box;21, Arba Minch, Ethiopia

**Keywords:** Children aged 6 to 59 months, Minimum dietary diversity, Ethiopia

## Abstract

**Background:**

Poor quality diets are the greatest obstacles to survival, growth, development, and learning in children**.** Dietary diversity is a major problem in developing countries including Ethiopia. For better feeding practices and focused interventions, it is essential to identify dietary diversity in children. In order to draw firm conclusions, previous studies in Ethiopia were unable to get a thorough picture of the dietary diversity among children under the age of five. Therefore, the purpose of this study was to identify minimum dietary diversity and associated factors among children under the age of five attending public health facilities in Wolaita Soddo town, Southern Ethiopia.

**Methods:**

An institution-based cross-sectional study design was used from February to March, 2021. The sample size of the study was 406. A simple random sampling was used to select the study participants. The minimum dietary diversity of the children was assessed by a standardized dietary assessment tool. The information was gathered using a standardized questionnaire that was administered by an interviewer. The collected data were entered into Epi Data 4.6 then exported to SPSS 26 for analysis. A multivariable logistic regression model was used to identify the independent predictors of the study. The statistical significance level was set at *P* < 0.05, and the degree of the association was measured using an AOR with a 95% CI.

**Results:**

A total of 399 participants were involved in this study. Nearly half (52.1%) of the study children met the minimum dietary diversity. The most popular foods were grains, roots, and tubers (79.2%), followed by dairy products (58.1%), vitamin A-rich fruits and vegetables, and grains, roots, and tubers (58.1%). Children whose mothers worked as daily workers had a lower likelihood of meeting the minimal dietary diversity requirement. However, children with separate eating plates from adults, households with food security, low monthly food expenditure, collaborative decision-making on household spending, birth intervals greater than 24 months, and health education on infant and young child feeding were more likely to achieve the minimal dietary diversity requirements.

**Conclusion:**

The proportion of the minimum dietary diversity was higher than in previous studies from Ethiopia. The minimum dietary diversity was higher than in previous studies from Ethiopia. Family planning for birth spacing, nutritional counseling on infant and young child feeding, and parent communication in a child's feeding are critical to improve dietary diversity in a child's feeding.

## Introduction

A dietary diversity refers to different food groups that are used to determine the variation and nutrient adequacy of diets [1–3]. Diversified diets are essential for children's nutritional demands, good growth, and development [3–5]. A minimum dietary diversity is defined as consuming at least four food groups out of the seven referenced food groups during the previous day [6]. For a child to grow holistically, it is essential to follow the right infant and young child feeding practices [3]. Exclusive breastfeeding for the first six months of life, continuing breastfeeding until age 2, introducing solid and semisolid foods at age 6 months, and gradually increasing the amount of food, variety, and feeding frequency as a child gets older are essential elements in feeding an infant and child [3, 6].

One of the biggest obstacles to children's survival, growth, development, and learning is poor nutrition [4, 7]. If their diets are not varied, infants and young children are at the danger of malnutrition, micronutrient deficiencies, morbidity, and mortality [3]. Insufficient meals are the most prevalent and significant cause of malnutrition [3, 8, 9].

Children under age 5 are disproportionately affected by the detrimental effects of malnutrition [8, 10, 11]. Malnutrition is a major concern among children in developing nations, particularly in Africa [7]. In South and East Africa, two out of every five children suffer from malnutrition [10, 11]. A country-wide study in Ethiopia found that 38% and 10% of children under the age of five had stunted growth and wasting, respectively [3]. In addition to this, the report [3] revealed that stunting affects children under the age of five, more severely between the ages of 6 and 23 months, and peaks at 24 to 35 months. Besides, malnutrition was a contributing factor in nearly 28% of child fatalities in Ethiopia [10, 11].

Studies showed that children with an adequate minimum dietary diversity less likely to be stunted and underweight [3, 12]. However, a lack of nutritional variety increases the morbidity and mortality of children [13]. The percentage of children that consume a minimally diverse diet stayed steady, at 21% in 2010 and 24% in 2020, according to a trend analysis of 50 countries [7]. However, the proportion of children that get the minimum acceptable diet varied by nation, culture, and region [3]. According to studies, the minimum dietary diversity in Ethiopia ranged from 8.5% to 59.9% [3, 14–17].

Furthermore, previous studies revealed that household food insecurity, individualized parental decision making on household expenditures, low monthly food expenditures, and a lack of health education on how to feed infants and young were negatively associated with the minimum dietary diversity [16–24].

The government of Ethiopia developed food-based dietary guidelines to promote healthy eating practices and lifestyles, providing general guidance on foods, food groups, and dietary patterns [9]. Furthermore, the Ethiopian government developed a national nutrition strategy as well as several initiatives to accelerate the reduction of child malnutrition [3]. Moreover, for better feeding practices and focused interventions, it is essential to identify dietary diversity in children [6].

Prior studies in Ethiopia [13–17] have investigated dietary diversity between the ages of 6 and 23 months. Little is known about children between the ages of 24 and 59 months due to the failure of earlier research to provide a comprehensive picture of children under the age of five. Thus, it is critical to determine the minimum dietary diversity among children under the age of five. Therefore, the purpose of this study was to identify minimum dietary diversity and associated factors among children under the age of five attending public health facilities in Wolaita Soddo town, Southern Ethiopia.

## Methods and materials

### Study area and period

The study was carried out in Wolaita Soddo town, which is an administrative town in the Wolaita zone in southern Ethiopia. The town is situated 152 km southeast of Hawassa, the regional center of the Southern Nations, Nationalities, and Peoples Region, and 327 km south of Addis Ababa. There was one teaching and referral hospital, one private hospital, three health centers, and thirteen private clinics in the town. The three study health centers offer services for adult outpatients, chronic disease clinics, reproductive health services or youth-friendly services, laboratory services, and pharmacy services, as well as pediatric and maternity health care [under five OPD, immunization, family planning, and delivery]. The teaching and referral hospital serves as a referral hospital in the Wolaita Zone, offering outpatient and inpatient services as well as pediatric, adult, and neonatal intensive care units, adult medical and surgical wards, and pharmacy and laboratory services. The study was conducted from June to July, 2021.

### Study design

An institution based cross-sectional study design was employed.

### Source population

All children aged 6 to 59 months who received health services in public health facilities in Wolaita Soddo town.

### Study population

Children aged 6 to 59 months who attended maternal and child health services and fulfilled the inclusion criteria.

### Inclusion and exclusion criteria

#### Inclusion criteria

All children aged 6 to 59 months who attended maternal and child health services during the data collection period, as well as children accompanied by their mother, father, or caregiver.

#### Exclusion criteria

Children who were critically ill, with known chronic diseases like DM and chronic heart diseases, and mothers or caregivers who were ill.

### Sample size and sampling technique

The sample size was calculated using a single population proportion with the following assumptions: the prevalence of a minimum dietary diversity, 59.9% from Addis Ababa ( [15] and a 95% confidence level with a 5% margin of error. Accordingly, the calculated sample size, with the consideration of a 10% non-response rate, was 406. This size was chosen as the final sample size because it gives a better sample size than the other calculated sample sizes.

### Sampling technique and procedures

**Fig. 1:**
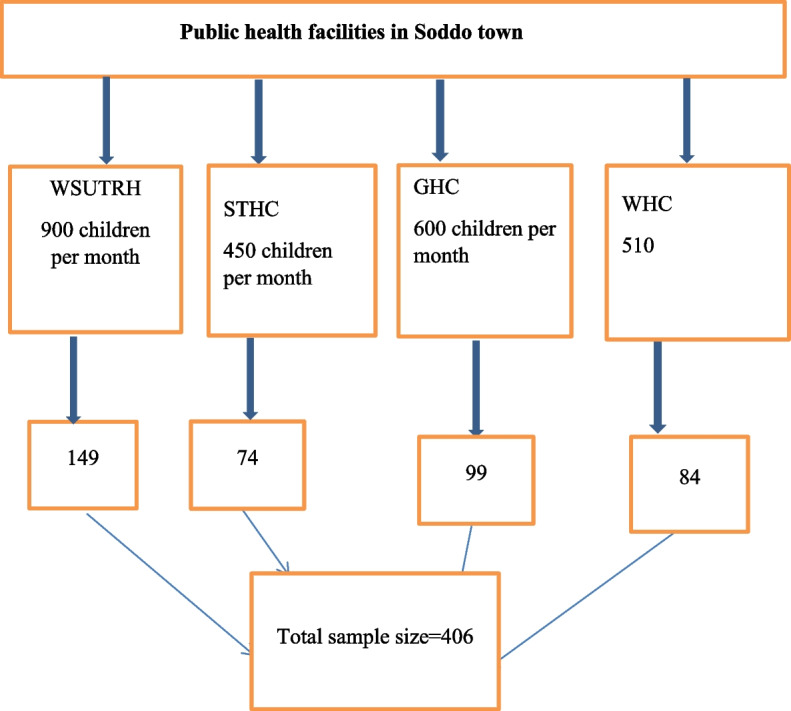
Schematic presentation of sampling procedure of the study, 2021

First, all the public health facilities in the town were selected for the study. Then, the computed sample size was proportionally allocated to the town's health facilities based on the average monthly flow of the children. Finally, study participants were selected using a computer-generated simple random sampling method as shown in Fig. 1.

### Data collection instruments and procedures

A pre-tested, interviewer-administered questionnaire was used to collect data from mothers, fathers, and child caretakers. Data collection tool was prepared after reviewing related literature [15–18]. The minimum dietary diversity of the children was assessed using a standardized dietary assessment tool [6]. The Food and Nutrition Technical Assistance (FANTA) Household Food Insecurity Access Scale Measurement Tool was used to assess the food security of households [25]. The questionnaire was translated into Amharic and the local language (Wolaittato) for fieldwork purposes and then translated back into the English language to check its consistency. The questionnaire contains four parts: socio-demographic characteristics of the children; utilization of child and maternal health services; household food security; and dietary diversity. The minimum of dietary diversity was assessed by asking the mothers or fathers or caretakers whether the child consumed food from the seven food groups on the previous day of the survey. The data were collected by ten trained nurses and supervised by four supervisors.

### Study variables

#### Dependent variable

Minimum dietary diversity among under-five children.

#### Independent variable

##### Socio-demographic characteristics

Parental occupation, education, family size, household income, food security, and family decision-making on household expenditures.

##### Child characteristics and maternal health service utilization

Age, sex, birth interval, and birth order, recurrent illnesses, antenatal care (ANC), post-natal care (PNC), immunization status, place of delivery, and health education on child feeding.

### Operational definitions

#### Minimum dietary diversity

The proportion of children aged 6 to 59 months who consumed at least four food groups out of the seven referenced food groups during the previous day of the study [6]. The seven food groups are: (1) grains, roots, and tubers; (2) legumes and nuts; (3) dairy products; (4) flesh foods (meats/fish/poultry); (5) eggs; (6) vitamin A-rich fruits and vegetables; and (7) other fruits and vegetables.

#### Food security

The state of having sufficient food at all times to meet dietary needs for a productive and healthy life [25].

### Data quality management

Training was given for the supervisors and data collectors on the purpose of the study, the techniques of data collection, and data recording. A pre-test was conducted on 5% of the total sample size outside of the study area (Boditi primary hospital). Based on the result of the pre-test, necessary modifications and corrections were made. The supervisors and investigators followed the data collection process on a daily basis. To ensure the quality of the data, each questionnaire was checked for consistency and completeness.

### Data processing and analysis

The data were entered into EpiData version 4.6, cleaned, and analyzed by SPSS version 26. The 4 scores of food security, i.e., "food secure," "mild food insecurity," "moderately food insecure," and "severely food insecure," were dichotomized into "food secure" and "food insecure." The results of the study were presented in text, tables, and graphs. A bivariate analysis was done to select the variables for a multivariate analysis. A multivariable logistic regression analysis was performed on the variables with a *P*-value < 0.25. Before adjusting in the multivariable analysis, the variables candidates for the multivariable analysis were checked for multi-collinearity using the variance inflation factor which ranged from [1.1—1.87]. A multivariable logistic regression analysis was done to identify the independent predictors of the minimum dietary diversity. The Hosmer-Lemes show test was used to assess the model's fitness [0.124]. *P*-values < 0.05 were considered statistically significant, and an adjusted odds ratio (AOR) with a 95% confidence interval was used to measure the degree of association.

### Ethical approval and consent to participate

Ethical clearance was obtained from the institutional review board at Addis Ababa University, the College of Health Sciences, and the School of Nursing and Midwifery with protocol number 70/21/SNM and meeting number: 01/2013EC. Written informed consent was obtained from the parents or legal guardians of the children, and the confidentiality of the information was maintained throughout the study. All methods and procedures utilized in this study were in conformity with the Declaration of Helsinki.

## Results

### Socio-demographic characteristics

This study included 399 participants in total, yielding a response rate of 98.27%. The majority (73.7%) of participants had a family size of 5 or above. The mean monthly income (in Ethiopian Birr) of study participants was 4395.49 (SD ± 2861.647) and the mean monthly food expenditure was 2249.53 (SD ± 1658.988) (in Ethiopian Birr) as shown below in Table 1.

**Table 1:** Socio-demographic characteristics of under-five children in public health facilities in Wolaita Soddo town, Southern Ethiopia, 2021(*n* = 399)

Variables	Category	Frequency	Percentage (%)
Mother education level	No formal	51	12.8%
	Primary	172	43.1%
	Secondary and above	176	44.1%
Father education	No formal	27	6.8%
	Primary	156	39.1%
	Secondary and above	216	54.1%
Mother occupation	Trader	57	14.3%
	Employee	97	24.3%
	Daily laborer	59	14.8%
	House wife	186	46.6%
Father occupation	Trader	107	26.8%
	Employee	139	34.8%
	Daily laborer	96	24.1%
	Farmer	57	14.3%
Residence	Urban	271	67.9%
	Rural	128	32.1%
Marital status	Married	356	89.2%
	Currently not married	43	10.8%
Family size in number	< 5	294	73.7%
	5 and above	105	26.3%
Separate feeding palate of a child from adults	No	163	40.9%
	Yes	236	59.1%

Where: Currently not married marital status includes single, widowed, and divorced

### Child characteristics and maternal health service utilization

One-third of the mothers, 135 (33.8%), have never attended follow-up antenatal care, as shown below in Table 2.

**Table 2:** Child characteristics and maternal health service utilization in public health facilities in Wolaita Soddo town, Southern Ethiopia, 2021(*n* = 399)

Variable	Category	Frequency	Percentage
Birth interval	1–23 months	115	28.8%
	> 24 months	284	71.2%
Birth order	1^st^-3^rd^	227	56.9%
	4^th^ and above	172	43.1%
Child age	< 24 months	190	47.6%
	≥ 24 months	209	52.4%
Child sex	Male	242	60.7%
	Female	157	39.3%
Antenatal care	No	135	33.8%
	Yes	264	66.2%
Post-natal care	No	137	34.3%
	Yes	262	65.7%
Immunization status of a child	Immunized	293	73.4%
	Not immunized	106	26.6%
Health education on how to feed children	No	140	35.1%
	Yes	259	64.9%
Place of delivery	Home	72	18%
	Health facility	327	82%

### Minimum dietary diversity

**Fig. 2:**
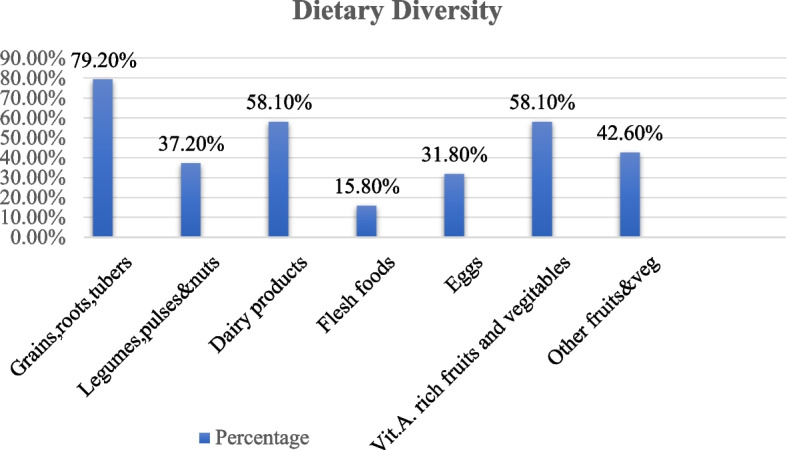
Dietary diversity among children in public health facilities in Sodo town, Southern Ethiopia

A total of 208 [(52.1%) % (47.6, 56.9) at 95% CI] children met the minimum dietary diversity. Grains, roots, and tubers (79.2%) were the most commonly consumed by the children, followed by dairy products, and vitamin-A-rich fruits and vegetables, each comprising 58.1%. Flesh foods (15.8%) and eggs (31.8%) were the least frequently consumed foods by the study children, as shown in Fig. 2.

### Multivariable analysis

The multivariable logistic regression analysis showed that children from food-secure households and children whose parents make decisions jointly on household food expenditures were more likely to meet the minimum dietary diversity standard. The children whose mothers worked as daily laborers, on the other hand, were 64.4% less likely to meet the minimum dietary diversity as shown below in Table 3.

**Table 3:** Multivariable regression analysis of the minimum dietary diversity and associated factors among under-five children attending public health facilities of Wolaita Soddo town, Southern Ethiopia, 2021(*n* = 399)

Variables	Minimum dietary diversity		COR (95%CI)	AOR (95%CI)
	**Yes**	**No**		
**Mothers’ education**				
No formal education	17(8.2%)	34(17.8%)	2.89(1.5,5.56)	1.97(0.62,6.20)
Primary	87(41.8%)	85(44.5%)	2.04(1.06,3.93)	2.02(0.72,5.66)
Secondary above	104(50%)	72(37.7%)	1.00	1.00
**Fathers Education**				
No formal education	13(6.3%)	14(7.3%)	1.34(.604, 3.00)	0.13(0.034,1 .53)
Primary education	75(36.1%)	81(42.4%)	0.97(0.44, 2.25)	0.33(0.101, 1.11)
Secondary and above	120(57.7%)	96(50.3%)	1.00	1.00
**Birth Interval**				
< 24 months	41(19.7%)	74(38.7%)	1.00	1.00
≥ 24 months	167(80.3%)	117(61.3%)	2.57(1.64, 4.04)	2.33(1.26,4.30) *
**Marital status**				
Married	201(96.6%)	155(81.2%)	6.66(2.89, 15.4)	0.98(0.317, 3.00)
Currently not married	7(3.4%)	36(18.8)	1.00	1.00
**Mothers’ occupation**				
Trader	31(14.9%)	26(13.6%)	1.58(0.87, 2.87)	0.54(0.207, 1.40)
Employee	81(38.9%)	16(8.4%)	6.7(3.64,12.34)	2.4(0.975, 5.90)
Daily laborer	16(7.7%)	43(22.5%)	0.49(0.26, 0.938)	0.35(0.128,0.099) *
House wife	80(38.5%)	106(55.5%)	1.00	1.00
**Father Occupation**				
Trader	52(25%)	55(28.8%)	1.50(0.78, 2.89)	1.102(0.38,3.192)
Employee	101(48.6%)	38(19.9%)	4.23(2.2, 8.10)	1.47(0.477, 4.52)
Daily laborer	33(15.9%)	63(33%)	0.83(0.422, 1.64)	1.32(0.49, 3.54)
Farmer	22(10.6%)	35(18.3%)	1.00	1.00
**Household food security**				
Secure	124(59.6%)	34(17.8%)	6.81(4.29,10.83)	3.63(1.95,6.76) **
Insecure	84(40.4%)	157(82.2%)	1.00	1.00
**Monthly food expenditure (in Ethiopian Birr)**				
Below mean	101(48.6%)	1489(77.5%)	1.00	1.00
Mean and above	107(51.4%)	43(22.5%)	3.64(2.360, 5.63)	2.29(1.05, 5.03) *
**Autonomy on household expenditure**				
Either father or mother	25(12%)	112(58.6%)	1.00	1.00
Jointly	183(88%)	79(41.4%)	10.38(6.24,17.2)	3.82(1.82,8.0) **
**Place of delivery**				
Home	28(13.5%)	44(23%)	1.00	1.00
Health institution	180(86.5%)	147(77%)	1.92(1.14, 3.24)	0.60(0.26, 1.42)
**Separate feeding plate**				
No	76(36.5%)	87(45.5%)	1.00	1.00
Yes	132(63.5%)	104(54.5%)	1.45(0.97,2.17)	3.09(1.69,5.68) **
**Health education on how to feed children**				
No	43(20.7%)	97(50.8%)	1.00	1.00
Yes	165(79.3%)	94(49.2%)	3.96(2.55,6.14)	3.13(1.72,5.69) **
**Fall sick in the past two weeks preceding data collection**				
No	36(17.3%)	25(13.1%)	1.39(0.80,2.41)	0.80(0.352, 1.83)
Yes	172(82.7%)	166(86.9%	1.00	1.00
**ANC follow-up**				
No	47(22.6%)	88(46.1%)	1.00	1.00
Yes	161(77.4%)	103(53.9%)	2.92(1.9,4.508)	1.21(0.61, 2.408)
**Post-natal care follow-up**				
No	59(28.4%)	78(40.8%)	1.00	1.00
Yes	149(71.6%)	113(59.2%)	1.74(1.148,2.64)	1.023(0.53, 1.95)

Currently not married marital status includes single, widowed, and divorced

*COR* Crude Odd Ratio, *CI* Confidence Interval, *AOR* Adjusted Odd Ratio

^*^Significant at: *p* < 0.05; **Significant at: *p* ≤ 0.001

## Discussion

Nearly half (52.1%) of the study children met the minimum dietary diversity. The result matched a report from Madagascar [26]. The result, though, was greater than what Ethiopian and Nigerian study reports had found [10, 16, 18, 27, 28]. Nonetheless, less than Peru [11], and Indonesia [29]. The disparities could be attributed to the fact that our study settings were urban areas, where there may be a better understanding of a varied diet, and the age difference (the majority of earlier studies focused on children between the ages of 6–23 months). This was supported by a report [3] that found that as children get older, their dietary diversity gradually increases. Additionally, the estimated dietary diversity may be affected by self-reported measurements; recall, and social desirability bias.

This study found that children from households with monthly food expenditures at or above the mean (measured in Ethiopian Birr) were twice as likely to meet the minimal dietary diversity standard as those below the mean. The result was in agreement with research from Ethiopia [15], Madagascar [26], Gambia [20], Algeria [19], Ghana [30], and Indonesia [29]. This is explained by the more varied diets that families with a higher socioeconomic level have, which contribute to children having acceptable nutritional status [3, 31, 32].

Furthermore, children from food-secure households were four times more likely than those from food-insecure households to meet the minimal dietary diversity requirements. The results were in line with study reports from Ethiopia [16, 33], Kenya [34],and Indonesia [29]. This could be explained by the fact that people in low socioeconomic positions have less access to a wide variety of foods, which leads to a diet that is poorer in quantity and quality. As a result, food-insecure households may be unable to meet their nutritional requirements [3, 31, 32].

In contrast, there was a 64.4% lower likelihood that the children whose mothers worked as daily laborers met the minimal dietary diversity requirement. The finding was in conformity with a study report [18]. However, a mother’s or parent’s was not associated, according to study reports [13, 14, 35]. This might be explained by the fact that people who work as daily laborers often have low socioeconomic status and cannot afford to feed their children a varied diet [31, 32].

Parents of children who jointly decided on household expenses had four times higher odds of meeting the minimum requirement for nutritional diversity than parents who individually decided on household expenses. It was consistent with a finding from Ethiopia [14]. This could be explained by the fact that joint decision-making enhances communication in a child's feeding.

The children with birth intervals of ≥ 24 months had twice higher odds of meeting the minimum dietary diversity as compared to their counterparts. The finding was supported by a study from Ethiopia [13]. This might be because birth spacing reduces the number of children in the household, allowing a child to eat a more varied diet.

Children who had separate meal plates from adults were three times more likely to have the minimum amount of dietary diversity. The finding verified with the finding from Ethiopia [21]. To reach a definitive conclusion, a small number of studies comparing a child's feeding plate to that of adults were reviewed.

The study found that children of mothers who received infant and young child feeding health education were three times more likely to meet the minimum dietary diversity standard. The finding of the study coincided with findings from Ethiopia [3, 17, 22], Tanzania [35], Malawi [23], and Guinea [24]. This is justified by the fact that the mothers who received infant and young child feeding education were more likely to feed the recommended diet [14].

### Limitation of the study

Since the study was cross-sectional in nature, it might be challenging to determine a cause-and-effect relationship. Besides, as the assessment of some variables relied on self-reported data, the study may be subject to recall bias and social desirability bias. Some variables, such as the area of the family's farmland, the goods they grow on it, the village's agro-ecology, and poultry production were not included. Moreover, given that our data were gathered from children who visited health facilities, illness may have an impact on a child's appetite.

## Conclusion

The proportion of the minimum dietary diversity was higher than in previous studies from Ethiopia. The minimum dietary diversity of children under the age of five was significantly correlated with the occupation of the mother, the monthly food expenditures, the household's food security status, the spacing between births, decision-making regarding household expenses, receiving health education regarding how to feed children, and keeping children's plates separate from adults. As a result, improving dietary diversity requires nutritional counseling on infant and young child feeding, family planning for birth spacing, improving household socio-economic status, and communication between a child's mother and father on child feeding. For better generalizability of the findings, future studies should take into account the limitations of this study.

## Data Availability

Data set used in this study will be available from corresponding author on reasonable request.
